# Overexpression of deubiquitinating enzyme USP28 promoted non-small cell lung cancer growth

**DOI:** 10.1111/jcmm.12426

**Published:** 2015-02-05

**Authors:** Lei Zhang, Biao Xu, Yong Qiang, Hairong Huang, Changtian Wang, Demin Li, Jianjun Qian

**Affiliations:** a Department of Cardiothoracic Surgery, Jinling Hospital, School of Clinical Medicine, Nanjing UniversityNanjing, Jiangsu Province, China

**Keywords:** USP28, NSCLC, prognosis, miR-4295

## Abstract

Non-small cell lung cancer (NSCLC) accounts for most lung cancer. To develop new therapy required the elucidation of NSCLC pathogenesis. The deubiquitinating enzymes USP 28 has been identified and studied in colon and breast carcinomas. However, the role of USP28 in NSCLC is unknown. The level mRNA or protein level of USP28 were measured by qRT-PCR or immunohistochemistry (IHC). The role of USP28 in patient survival was revealed by Kaplan–Meier plot of overall survival in NSCLC patients. USP28 was up or down regulated by overexpression plasmid or siRNA transfection. Cell proliferation and apoptosis was assayed by MTT and FACS separately. Potential microRNAs, which targeted USP28, were predicated by bioinformatic algorithm and confirmed by Dual Luciferase reporter assay system. High mRNA and protein level of USP28 in NSCLC were both correlated with low patient survival rate. Overexpression of USP28 promoted NSCLC cells growth and vice versa. Down-regulation of USP28 induced cell apoptosis. USP28 was targeted by miR-4295. Overexpression of USP28 promoted NSCLC cells proliferation, and was associated with poor prognosis in NSCLC patients. The expression of USP28 may be regulated by miR-4295. Our data suggested that USP28 was a tumour-promoting factor and a promising therapeutic target for NSCLC.

## Introduction

Non-small cell lung cancer (NSCLC) is thought to originate in lung epithelial cells, and comprises diverse histological subtypes including adenocarcinoma, bronchioloalveolar, squamous, anaplastic and large-cell carcinomas 1. NSCLC accounts for most lung cancer 2,3. Lung cancer incidence has peaked and declined in several region of the world but has yet to peak in many other parts of the world, particularly China 4. During the past 30 years, China has experienced a dramatic increase in the cigarette consuming, and the mortality of lung cancer has increased by 465% during the past 30 years. Most patients with advance NSCLC present with metastatic disease and, if left untreated, have a median survival after diagnosis of 4–5 months and a 1-year survival of less than 10% 5. Combination cytotoxic chemotherapy results in a modest increase in survival at the cost of significant toxicity to the patients 6,7. To develop new therapy required the elucidation of NSCLC pathogenesis.

The function of deubiquitinating enzymes (DUBs) to control substrate activity and/or abundance by removing covalently attached ubiquitin from proteins 8. The USP family in DUBs plays an important role in cellular processes and signalling pathways 9–15. High expression of USP28 was found in colon and breast carcinomas. More importantly, USP28 controls MYC stability through antagonizing the activity of the SCF^FBW7^ ubiquitin liagse complex, and that the stabilization of MYC by USP28 is required for proliferation of several tumour cells types and for inhibition of cell differentiation in colon carcinoma, however, the role of USP28 in lung cancer has not yet been determined 16. Here, we focused the role of USP28 in NSCLC, and tried to elucidate the involved mechanism.

MicroRNAs (miRNAs) inhibited mRNA translation or induce mRNA degradation in general by binding to the 3′untranslation region (3′UTR) of target mRNAs 17. Accumulating data showed that specific miRNAs were involved in early-stage NSCLC 18–22. We wondered, in NSCLC, USP28 could be targeted by some miRNAs.

Here, we studied the role of USP28 in NSCLC, and searched potential miRNAs which targeted USP28. We hoped our study might promote the elucidation the pathogenesis of NSCLC and provide potential therapy targets for further investigation.

## Materials and methods

### Patients

Surgical specimens from 70 NSCLC patients and matched normal control adjacent normal tissues were obtained postoperatively in 2009 from the Jinling Hospital, Nanjing University, School of Medicine. Tissues were obtained before chemotherapy and radiotherapy and were immediately frozen and stored at −80°C prior to qRT-PCR assay. Informed consent was obtained from each patient and the study was approved by the Ethics Committee of Nanjing University, Nanjing, China. All diagnoses were based on pathological and/or cytological evidence.

### Cell culture

Human normal lung fibroblast cell line MRC-5, and human NSCLC cell lines (SPC-A-1, LTEP-A-2, SK-MES-1, A549 and H1299), HEK293 comes from Cell Bank of Chinese Academy of Science (Shanghai, China). These cell lines were maintained in RPMI1640 (Invitrogen, Carlsbad, CA, USA) in the presence of 10% heat-inactivated foetal bovine serum, 100 IU/ml penicillin and 100 ug/ml streptomycin in a humidified 5% (v/v) atmosphere of CO_2_ at 37°C.

### RNA extraction and real-time q-PCR

RNA was extracted with Trizol reagent (Invitrogen) according to the manufacturer's protocol. The cDNA synthesis and real-time qPCR were subsequently performed with the Qiagen system as described detail in previous studies 23. Real-time quantitative PCR analysis was performed with standard protocols on an Applied Biosystem's (Burnsville, Minnesota, United States) 7500 HT sequence Detection System. MiR-4295 expression was assessed using a mirVana™ qRT-PCR miRNA Detection Kit (Ambion, Austin, Texas, USA). The primers were designed and synthesized by Shengong Company (Shanghai, China). Relative mRNA levels of USP28 were normalized to levels of the housekeeping gene GAPDH and calculated by the 2^−ΔΔCt^ method. The primers used are as follows: GAPDH (5′-CCATGTTCGTCATGGG-TGTGAACCA-3′ and 5′-GCCAGTAGAGGCAGGGATGATGTTG-3′) and USP28 (5′-ATCTTCAGGCTGCCATTGCT-3′ and 5′-CTAGCTGGAATGCGTCCTCT-3′).

### Immunohistochemistry

Immunohistochemistry (IHC) staining was described previously 24. Briefly, 4-μm thick sections were cut and anti-USP28 antibody (Sigma-Aldrich (St. Louis, MO, USA)) was applied. Subsequent counterstaining was performed with haematoxylin. Immunostaining results for USP28 were evaluated using a semi-quantitative scoring system as described previously 25, which calculated the staining intensity and the percentage of positive cells. IHC staining was scored according to the following criteria: −, 0–10% of the nucleated cells stained, +, 10–40% stained, ++; 40–70% stained and +++, 70–100% stained. USP28 expression was considered to be observed when score≧ +. Alternatively, IHC score of USP28 expression was (− ∼ +) and (++ ∼ +++), which represented low and high expression, respectively.

### USP28 overexpression plasmid, siRNA, miRNAs and cells transfection

USP28 overexpression plasmid (pcDNA3.1-USP28), siRNA and control were designed and constructed by Santa Cruz Biotechnology Delaware Ave Santa Cruz, CA. 95060, USA. miR-NC, miR-16, miR-4295, miR-363-3p, miR-301a, miR-301b, miR-453, miR-3066, miR-130a and miR-130b were constructed by Shengong Company. Transfections were performed with Lipofectamine 2000 reagent (Invitrogen) according to the manufacturer's protocol.

### MicroRNAs prediction

TargetScanHuman (http://www.targetscan.org/vert_61/) 26–29 is applied to identify which miRNAs target USP28.

### MTT assay

For MTT assay, 5 × 10^3^ cells per well were seeded in triplicate in a 96-well plate with complete growth medium. Cells were counted over 5 days using the MTT assay (Promega, Fitchburg, WI, USA) as described previously 30–32. The data were measured by Microtiter plate reader 570-nm filters (Promega).

### Apoptosis assay

Cells were labelled with Annexin V-FITC and propidium iodide (PI) using an apoptosis detecting kit (Invitrogen, Burlington, ON, Canada) following the manufacturer's instructions. Then sample were analysed by FACS assays (Becton Dickinson, San Jose, CA, USA) 33.

### USP28 3′UTR reporter analysis

The 3′UTR reporter plasmids (RL-USP28) were constructed by Shengong Company. Mutation in the miR-4295 seed regions of the USP28 3′UTR were generated using QuikChang Multi site-directed mutagenesis kit (Stratagene). RL reporter plasmids (3.6 fmol) and pGL3-control (500 ng for normalization; Promega) were transfected with Lipofectamine 2000 (Invitrogen) into HEK293 cell (6 × 10^4^ cells per well). These Cells were collected after 48 hrs for assay using the Dual Luciferase reporter assay system (Promega) 34.

### Statistical analysis

Data were presented as the mean ± SD from at least three independent experiments. The difference between groups was analysed using two-tailed Student's *t*-test when only two groups were compared. The Wilcoxon matched-pairs signed rank test was used to determine if there was a statistically significant difference in the expression of USP28 between matched pairs. The difference between groups was analysed using anova when three or more than three groups were compared. Correlation analysis was performed by two-tailed Person's correlation coefficient analysis. Patient survival was determined by Kaplan–Meier analysis. Statistical analyses were performed with Statistics Package for Social Science, (SPSS), Armonk, NY, USA, (version 17.0). *P* < 0.05 was considered significantly different.

## Results

### High mRNA level of USP28 in NSCLC reduced patients survival

**Fig 1:**
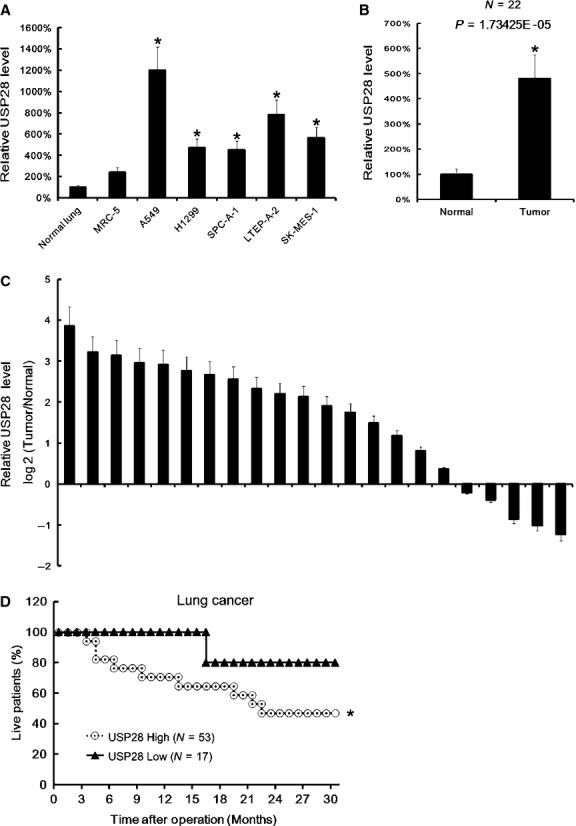
High mRNA level of USP28 in NSCLC was correlated with low patients survival. After total RNA extraction, USP28 mRNA expression in normal lung tissues, MRC-5, A549, H1299, SPC-A-1, LTEP-A-2 and SK-MES-1were assayed by qRT-PCR. The USP28 expression in normal lung tissues were arbitrarily defined as 100%. Data are mean ± SD of three separate experiments (A). The mean expression of USP28 in NSCLC tissues and adjacent normal tissues were assayed by qRT-PCR. The USP28 mean expression in normal lung tissues were arbitrarily defined as 100%, Data are mean ± SD of 22 samples, *N* = 22 (B). The USP28 expression in tumour tissues and in adjacent normal tissues were assayed by qRT-PCR. The difference of USP28 expression between in tumour tissues and in adjacent normal tissues in each patient were compared. There are 17 of 22 cases, which showed a higher USP28 expression in tumour tissues than in adjacent normal tissues. (C). The Kaplan–Meier plot of overall survival in NSCLC patients post-operation according to the USP28 mRNA expression. The mean value of all 70 NSCLC tissues was chosen as the cut-off point for separating USP28 high expression (*n* = 53) from USP28 low expression cases (*n* = 17)(D). *P < 0.05

We firstly assayed USP28 level in NSCLC cell lines, and found that NSCLC cell lines (A549, H1299, SPC-A-1, LTEP-A-2 and SK-MES-1) showed a high USP28 (Fig.1A). And then USP28 level in 22 NSCLC tissues was assayed by qRT-PCR. We found that the mean expression of USP28 in NSCLC tissues was higher than control (Fig.1B). In the 22 NSCLC specimens, there were 17 case with higher USP28 expression in tumour tissues (Fig.1C). Next, we investigated the relationship between USP28 expression and patient survival and found high USP28 expression was correlated with low patient survival rate, and the mean value of all 70 NSCLC tissues was chosen as the cut-off point for separating USP28 high expression (*n* = 53) from USP28 low expression cases (*n* = 17; Fig.1D).

### High protein level of USP28 in NSCLC reduced patients' survival

**Fig 2:**
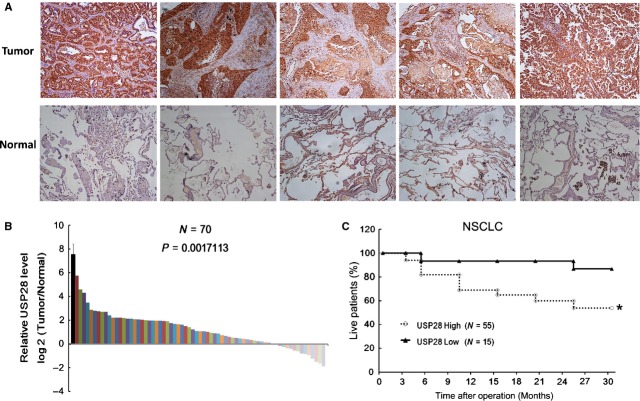
Expression of USP28 in NSCLC tissues. Representative images of USP28 immunohistochemistry staining in NSCLC tissues and matched normal adjacent tissues (A). The USP28 protein expression in 70 pairs tissues were digitalized and compared, there are 55 of 70 cases, which showed higher USP28 expression in tumour tissues than in adjacent normal tissues (B). 70 NSCLC patient had been followed-up for 30 months, deceased cases were recorded. The Kaplan–Meier curves were drawn according to the USP28 expression (C); *P < 0.05.

To further investigate the role of USP28, we assayed USP28 protein expression in NSCLC tissues by IHC. We found that NSCLC tissues showed higher USP28 protein levels than adjacent normal tissues (Fig.2A). Next the USP28 protein expressions were scored according to the criteria mentioned in Materials and Methods part. We found in the majority NSCLC tissues, USP28 expression were higher (Fig.2B). Similarly, we investigated the relationship between USP28 protein expression and patient survival and found high USP28 protein expressions were correlated with low patient survival rate (Fig.2C).

### Up-regulation of USP28 promoted NSCLC cells proliferation

**Fig 3:**
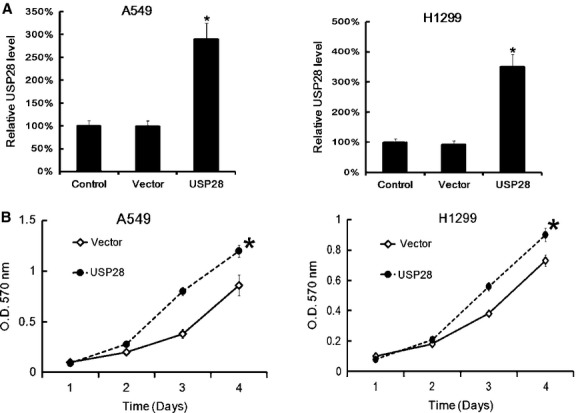
USP28 overexpression plasmid transfection promoted NSCLC cells proliferation. A549 and H1299 (6 × 10^5^ cells/well) were transfected with pcDNA3.1-USP28 or empty vector separately. 48 hrs later, the USP28 expression was assayed by qRT-PCR. Data were normalized to GAPDH. The USP28 expression in normal lung control were arbitrarily defined as 100%. Data are mean ± SD of three separate experiments (A). After pcDNA3.1-USP28 transfection, the cells proliferation was assayed by MTT at the indicted time-point. Data are mean ± SD of three separate experiments (B); *P < 0.05.

Next we up-regulated the USP28 expression by plasmid transfection, and found that 48 h after transfection, the USP28 expression were higher than control in A549 and H1299 (Fig.3A). Then cells proliferation was assayed by MTT. We found that up-regulation of USP28 in NSCLC cell lines promoted cells proliferation (Fig.3B).

### Down-regulation of USP28 promoted NSCLC cells proliferation and induced cells apoptosis

**Fig 4:**
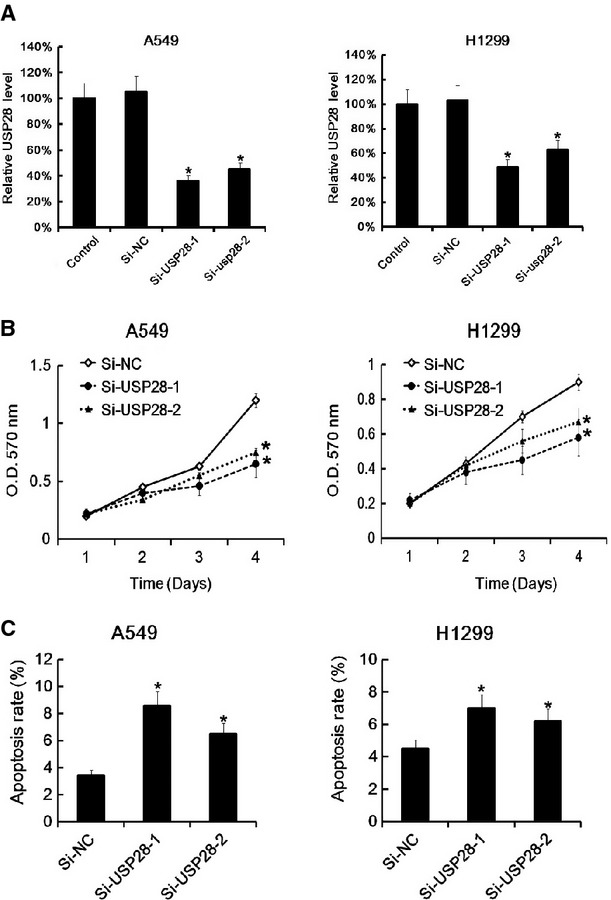
USP28 siRNA transfection promoted NSCLC cells proliferation and induced cells apoptosis. A549 and H1299 (6 × 10^5^ cells/well) were transfected with siRNA for USP28 or negative control separately. 48 hrs later, the USP28 expression was assayed by qRT-PCR. Data were normalized to GAPDH (A). After siRNA transfection, the cells proliferation was assayed by MTT at the indicted time-point. Data are mean ± SD of three separate experiments (B). The cell apoptosis rate after 48 hrs siRNA transfection in A549 and H1299 was assayed by Annex V PI-FACS analysis. The apoptosis rate in negative control group was arbitrarily defined as 100%. Data are mean ± SD of three separate experiments (C); *P < 0.05.

Then we down-regulated the USP28 expression by siRNA (si-USP28). 48 hrs after siRNA transfection, The USP28 expression was significantly down-regulated (Fig.4A). As expected, MTT assay showed that both two siRNAs reduced A549 and H1299 cells proliferation (Fig.4B). Moreover, FACS apoptosis analysis indicated that down-regulation of USP28 expression by siRNA increased the NSCLC cells apoptosis rate (Fig.4C).

### USP28 was targeted by miR-4295

**Fig 5:**
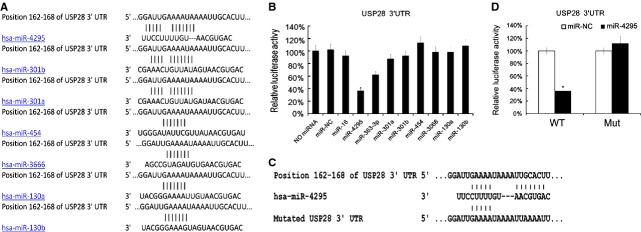
USP28 3′UTR reporter analysis revealed that miR-4295 targeted USP28. Which miRNAs targeted USP28 were predicated by TargetScanHuman, the binding site between potential miRNAs and USP28 were shown (A). The RL reporter plasmids (RL-USP28) and miR-NC, miR-16, miR-4295, miR-363-3p, miR-301a, miR-301b, miR-453, miR-3066, miR-130a and miR-130b were cotransfected into HEK293 cells separately, along with a firefly luciferase reporter (pGL control) for normalization. Luciferase activities were measured after 48 hrs. Then the ratio of RL activity of firefly luciferase activity in treated group were calculated and compared with the ratio in negative control group (which was arbitrary defined as 100%). Data are mean ± SD of three separate experiments (B). Position of 162-168 of USP28 3′UTR were mutated (C). The RL reporter plasmids with mutated USP28 3′UTR and miR-4295 were cotransfected into HEK293 cells, along with a firefly luciferase reporter (pGL control) for normalization. Luciferase activities were measured after 48 hrs. Then the ratio of RL activity of firefly luciferase activity in miR-4295 treated group were calculated and compared with the ratio in miR-NC group (which was arbitrary defined as 100%). Data are mean ± SD of three separate experiments (D); *P < 0.05.

Next, we tried to find which miRNAs targeted USP28. Bioinformatics algorithms were applied to predicate the potential miRNAs. Based on their potential relevance to NSCLC and novelty, parts of predicated miRNAs were chosen and shown in Figure5A. Next the 3′UTR of the USP28 were cloned into luciferase reporter plasmids. 9 miRNAs and the reporter plasmids were cotransfected into HEK293 cells. We found that miR-4295 reduced the luciferase activity mostly (Fig.5B). To confirm the role of miR-4295, we mutated USP28 3′UTR as Figure5C indicated. Mutated USP28 3′UTR was cloned into luciferase reporter plasmids, and then miR-4295 and reporter plasmids with mutated USP28 3′UTR were cotransfected into HEK293 cells. We found that miR-4295 did not reduce the luciferase activity when transfected with mutated USP28 3′UTR (Fig.5D). Thus, our data indicated that USP28 was targeted by miR-4295.

## Discussion

In this study, we demonstrated the role of USP28 in NSCLC. Significantly, we proved that high USP28 expression in mRNA level and protein level were both correlated with low patient survival rate. Thus, USP28 expression in NSCLC may be treated as a predictor for the prognosis of NSCLC. We also found that up-regulation of USP28 promoted NSCLC cells proliferation and vice versa. Our data showed that USP28 on proliferation and apoptosis were rather minor (4–8% change maybe). It is surprising that these minor influences could cause an effect on patient survival. We guessed that Although USP28 on proliferation and apoptosis were rather minor, cell proliferated exponentially *in vivo*. Thus, minor USP28 difference caused an effect on patients survival during a long time (30 months).

Previous study demonstrated that depletion of USP28 inhibited both cell-cycle and cell growth through regulation of MYC abundance 16. Another study revealed that in NSCLC, overexpression of USP14 promoted cells growth through the accumulation of β-Catenin 35. USP28 was also involved in DNA-damage-induced ubiquitination and deubiquitination as the major regulator of the DNA-damage response for Chk, 53BP1 and a number of other proteins in the DNA-damage checkpoint pathway, including several mediators, such as Mdc1, Claspin and TopBP1 36. It seemed USP28 was involved in multiple-pathway. Which pathway USP28 exploited in NSCLC needed further investigation. In the next study, we will compare the gene profiles between NSCLC cell lines before and after USP28 up- or down-regulation.

Besides, we proved that in A549 and H1299, up-regulation of USP28 promoted NSCLC cells proliferation. Interestingly, A549 expressed wild-type p53 and H1299 was p53-deficient. Previous study indicated that USP28 regulated Chk2-p53-PUMA pathway, which is a major regulator of DNA-damage-induced apoptosis 36.

Our data indicated that miR-4295 may have targeted USP28 in NSCLC. This may be the first report about miR-4295 in NSCLC. We guessed that the level of miR4295 was related with the survival of NSCLC patients. We will investigate the role of miR4295 in further study.

In conclusion, our data indicated that high expression of USP28 in NSCLC promoted tumour cells proliferation, and miR-4295 may target USP28. Our study is a precursor study of USP28 and miR-4295, and may provide a potential target for further study.

## Conflicts of interest

The authors have declared that no competing interests exist.
